# The causal effect of hypertension, intraocular pressure, and diabetic retinopathy: a Mendelian randomization study

**DOI:** 10.3389/fendo.2024.1304512

**Published:** 2024-02-06

**Authors:** Xiao-Fang Wang, Xiao-Wen Zhang, Ya-Jun Liu, Xin-Yu Zheng, Meng-Ru Su, Xing-Hong Sun, Feng Jiang, Zhi-Nan Liu

**Affiliations:** ^1^ Department of Ophthalmology, Nanjing Drum Tower Hospital, The Affiliated Hospital of Nanjing University Medical School, Nanjing, China; ^2^ Department of Endocrinology, Nanjing Drum Tower Hospital, The Affiliated Hospital of Nanjing University Medical School, Nanjing, China; ^3^ Department of Ophthalmology, Changzhou Third People’s Hospital, Changzhou Medical Center, Nanjing Medical University, Changzhou, China

**Keywords:** diabetic retinopathy, hypertension, IOP, GWAS, Mendelian randomization

## Abstract

**Background:**

Previous research has indicated a vital association between hypertension, intraocular pressure (IOP), and diabetic retinopathy (DR); however, the relationship has not been elucidated. In this study, we aim to investigate the causal association of hypertension, IOP, and DR.

**Methods:**

The genome-wide association study (GWAS) IDs for DR, hypertension, and IOP were identified from the Integrative Epidemiology Unit (IEU) Open GWAS database. There were 33,519,037 single-nucleotide polymorphisms (SNPs) and a sample size of 1,030,836 for DR. There were 16,380,466 SNPs and 218,754 participants in the hypertension experiment. There were 9,851,867 SNPs and a sample size of 97,465 for IOP. Univariable, multivariable, and bidirectional Mendelian randomization (MR) studies were conducted to estimate the risk of hypertension and IOP in DR. Moreover, causality was examined using the inverse variance weighted method, and MR results were verified by numerous sensitivity analyses.

**Results:**

A total of 62 SNPs at the genome-wide significance level were selected as instrumental variables (IVs) for hypertension-DR. The results of univariable MR analysis suggested a causal relationship between hypertension and DR and regarded hypertension as a risk factor for DR [*p* = 0.006, odds ratio (OR) = 1.080]. A total of 95 SNPs at the genome-wide significance level were selected as IVs for IOP-DR. Similarly, IOP was causally associated with DR and was a risk factor for DR (*p* = 0.029, OR = 1.090). The results of reverse MR analysis showed that DR was a risk factor for hypertension (*p* = 1.27×10^-10^, OR = 1.119), but there was no causal relationship between DR and IOP (*p* > 0.05). The results of multivariate MR analysis revealed that hypertension and IOP were risk factors for DR, which exhibited higher risk scores (*p* = 0.001, OR = 1.121 and *p* = 0.030, OR = 1.124, respectively) than those in univariable MR analysis. Therefore, hypertension remained a risk factor for DR after excluding the interference of IOP, and IOP was still a risk factor for DR after excluding the interference of hypertension.

**Conclusion:**

This study validated the potential causal relationship between hypertension, IOP, and DR using MR analysis, providing a reference for the targeted prevention of DR.

## 1 Introduction

According to the report of the International Diabetes Federation, approximately 400 million people have diabetes currently, and the prevalence rate of diabetes will be close to 600 million by 2035 (1, 2). Among the microvascular complications of diabetes, diabetic retinopathy (DR) is reported to be the most common cause of visual impairment in adults of working age (3). Additionally, some patients with diabetes mellitus type 2 (T2DM) still acquire javascript:;DR after 6.5–13.3 years even if blood sugar is strictly controlled (4, 5). Identifying the risk factors of DR is an important part of implementing preventive interventions to deal with the growing threat.

Since hypertension is a condition that can be controlled, an increasing number of observational epidemiological studies are focusing on how it impacts DR. Based on the existing research, the relationship between the outcomes of hypertension-related factors and the emergence of DR is controversial and inconsistent. Based on the findings of the Large Prospective UK Prospective Diabetes Study, DR is associated with systolic blood pressure (SBP) in patients with T2DM (6). However, the onset and progression of DR in patients with T2DM were not correlated with baseline systolic and diastolic blood pressure in the same study (7). Some studies have reported that SBP and hypertensive retinopathy are the primary predictors of DR in patients with T1DM solely and have no impact on T2DM (8). In a community-based DR screening study of Chinese adults with T2DM, it is necessary to reduce SBP (<140 mmHg) and glycosylated hemoglobin (HbA1c) (<7.0%) levels in combination rather than alone, which is believed to be related to a significant reduction in the possibility of DR (9).

An increasing number of studies are focusing on the relationship between DM/DR/intraocular pressure (IOP). DM is believed to easily damage the optic nerve via retinal vascular destruction and imbalance and various neurodegenerative mechanisms and destroy the trabecular meshwork route of water outflow, further causing high IOP and optic nerve injury (10–12). A cohort study has revealed that among patients with diabetes, patients with glaucoma and/or high IOP were more likely to develop DR within 5 years (13). However, Gangwani et al. conducted a cross-sectional study on 470 patients with DR and demonstrated that there was no correlation between DR and glaucoma (14). Nevertheless, there may be a longitudinal association between DR, glaucoma, and/or high IOP in the DR population.

To recapitulate, the causal relationship between IOP and DR remains elusive. Although the potential confounding factors are strictly controlled, residual unmeasured confounding factors can still affect the analysis of observational studies. Both the causal relationship between potential risk factors and the risk of DR and the suggestion for preventive treatment cannot be established solely based on observational data. Mendelian randomization (MR) analysis used genetic predictors as instrumental variables (IVs) to examine the causal relationship between risk factors and diseases. Since genetic variation is randomly distributed at conception, each IV is considered an alternative to randomized controlled trials (RCTs), which can prevent various deviations present in traditional observational studies.

MR analysis of DR exhibits that intestinal flora and adiponectin are also reported to have a direct causal relationship with DR in addition to traditional unhealthy habits such as smoking and drinking (15–18). However, whether there is a direct causal relationship between hypertension, IOP, and DR remains elusive. Our study for the first time provides direct evidence of the causal relationship between hypertension and IOP as independent risk factors and their combined effects on the risk of DR via MR analysis.

## 2 Materials and methods

### 2.1 Outcome source and data pre-processing

**Figure 1:**
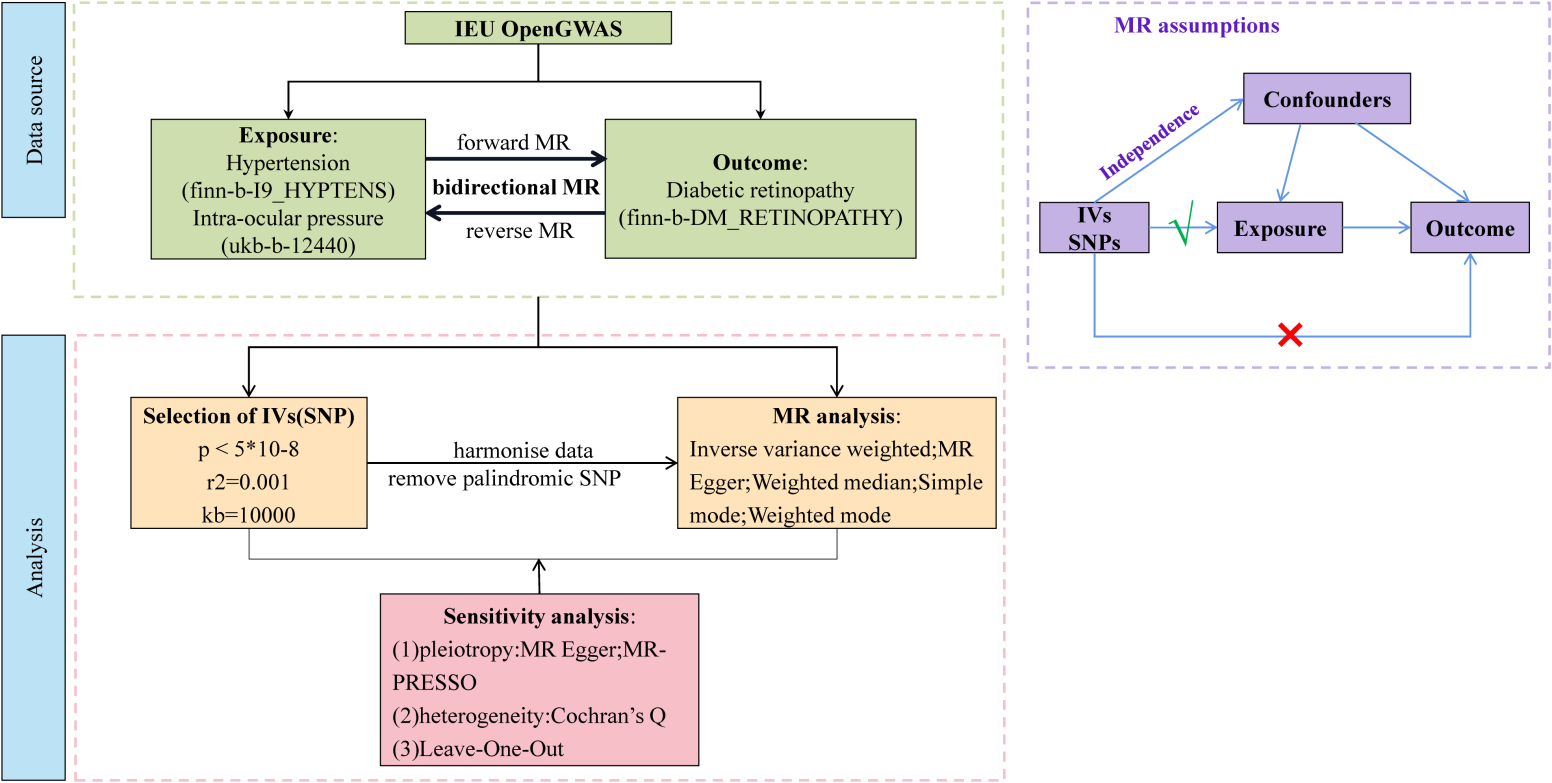
Overview of Mendelian randomization (MR) analyses process and basic assumptions.

Our study was a bidirectional MR analysis. The DR dataset was gathered from the genome-wide association studies (GWAS) database (https://gwas.mrcieu.ac.uk/). There were 33,519,037 single-nucleotide polymorphisms (SNPs) and a sample size of 1,030,836 for DR. There were 16,380,466 SNPs and a sample size of 218,754 for hypertension. There were 9,851,867 SNPs and a sample size of 97,465 for IOP. Genetic variants for hypertension and IOP were derived from GWAS datasets with 408,442 and 307,638 individuals each, respectively. The overview of this study’s design is presented in **Figure 1**. Since the data we used were based on published studies and public databases, no additional ethical approval from an institutional review board was required.

### 2.2 Selection of eligible instrumental variable

This MR study was based on the following three core assumptions: (1) there is a robust association between IVs and exposure; (2) the IVs are independent of confounders; and (3) the IVs can only affect the outcome via exposure without other pathways. Therefore, to meet the first assumption, the SNP set was restricted to be directly associated with the exposure at the genome-wide significant *p*-value threshold at *p<* 5 × 10^-8^ as potential instruments. Reading of exposure factors and filtering of IVs were done using the two-SampleMR R package (version 0.5.6) and the extract instrument function (19). The strength of IVs was assessed by calculating the *F*-statistic using the formula $\text{F=}\frac{R^{2}\times (N-1-K)}{{(1-R}^{2})}$ , where *R^2^* represents the proportion of variance in the exposure explained by the genetic variants, *N* represents sample size, and *K* represents the number of instruments. If the corresponding *F*-statistic was > 10, it was considered that there was no significant weak instrumental bias (20).

### 2.3 MR analysis

Independent SNPs were clumped at a threshold of linkage disequilibrium (LD) at *r* ^2 =^ 0.001 within the window of 10 megabase pairs; eligible IVs were selected to prevent double counting and biased causal effect estimates. Furthermore, the IVs were extracted from the outcome trait and harmonized in both exposure and outcome GWAS. In this step, palindromic SNPs with intermediate allele frequency were removed.

We performed a total of two univariable MR analyses using summary statistics from a GWAS to investigate the bidirectional association between hypertension, IOP, and DR. The forward MR analyses considered hypertension and IOP as the exposure and DR as the outcome, while the reverse MR analyses considered DR as the exposure and hypertension and IOP as the outcome. Five methods—MR Egger, weighted median, inverse variance weighted (IVW), simple mode, and weighted mode—were used to complete the univariable MR analysis. IVW was utilized as the primary MR analysis method, which yielded reliable results if there was no horizontal pleiotropy, with the other methods serving as secondary methods. We used multivariable MR analysis, an extension of univariable MR that enables concurrent detection of causative effects of multiple risk factors, to investigate the direct effects of hypertension and IOP on DR. The IVW method was used for MR analysis, which provided a consistent estimate of the relationship between exposure and the risk of outcomes when IVs were not pleiotropic. The SNPs used to conduct multivariable MR were combinations of IVs of each exposure. Cochran’s *Q* statistics was performed to assess the heterogeneity across individual SNPs.

Sensitivity testing was performed using the following three methods: (1) the heterogeneity test mr_heterogeneity, where a *Q* value >0.05 indicates that there is no heterogeneity; (2) the horizontal pleiotropy test Horizontal pleiotropy, where a *p*-value >0.05 indicates that there is no horizontal pleiotropy; (3) the Leave-One-Out method, which aims at determining whether there are outlier values for the effect of each SNP.

## 3 Results

### 3.1 Forward univariable MR analysis of hypertension, IOP, and DR

**Figure 2:**
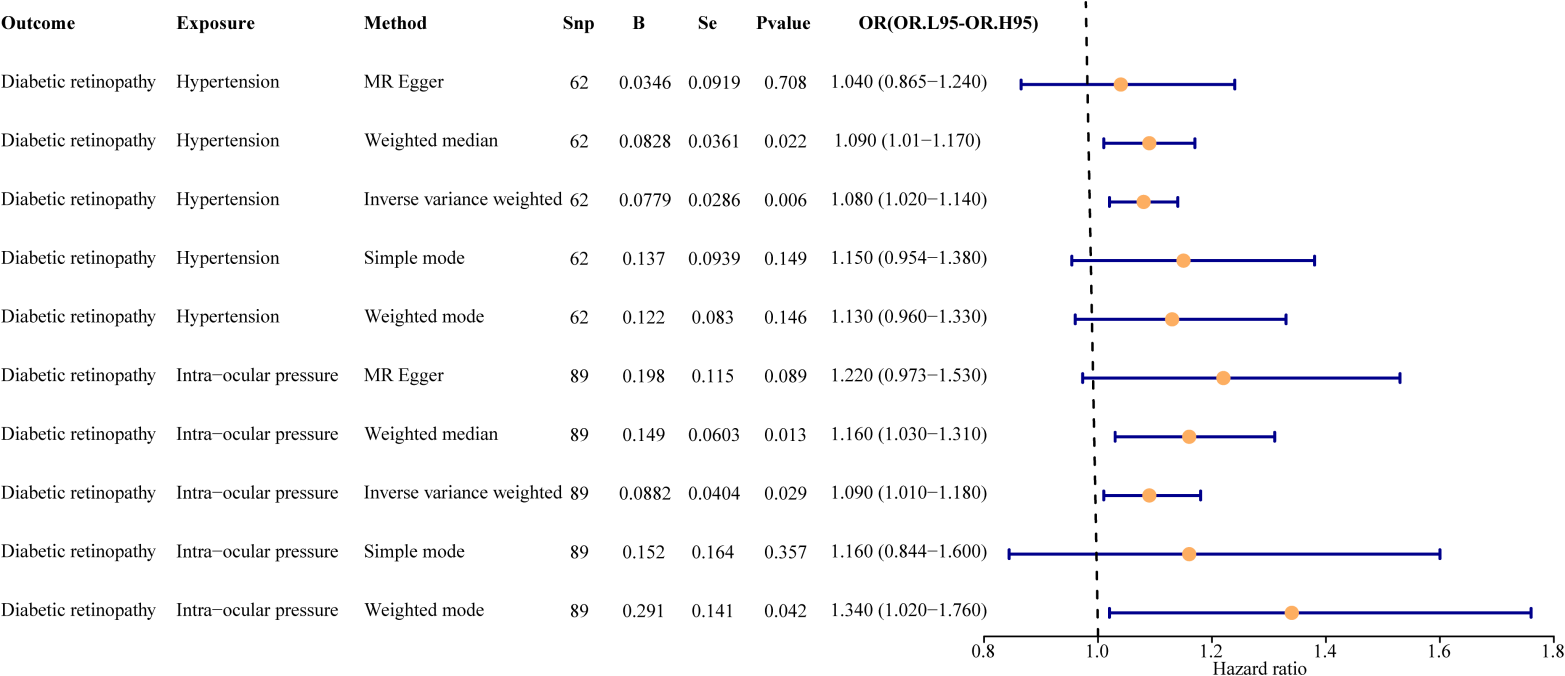
Univariate result forest map. The orange circle indicates that hazard ratio (HR) > 1, and the line segments on either side of the circle are 95% confidence intervals (CI) for HR values.

**Figure 3:**
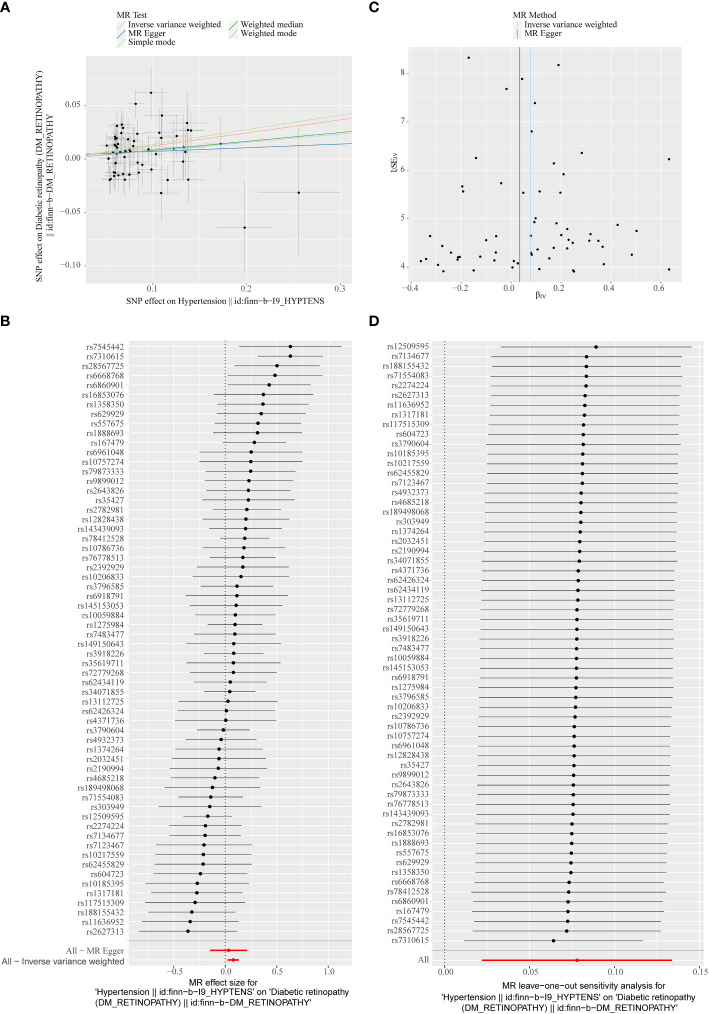
**(A)** Scatter plots of the MR analyses for the association of hypertension and the risk of diabetic retinopathy (DR). The colored lines represent fitting result of different algorithms. Error bars indicate 95% CI. **(B)** Forest plot of MR results of hypertension effect on DR. **(C)** Funnel plots of MR estimates for the effect of hypertension on the risk of DR. **(D)** Leave-One-Out plot for sensitivity test for the effect of hypertension on DR.

In the primary analysis, a total of 62 SNPs at the genome-wide significance level were selected as IVs for hypertension-DR (**Supplementary Table 1**). The results of univariable MR analyses suggested a causal relationship between hypertension and DR and hypertension as a risk factor for DR (*p* = 0.006, OR = 1.080). The weighted median method result was similar to that of IVW (*p* = 0.022, OR = 1.090, **Figure 2**). The scatter plot suggested that the slope of the lines for all five methods was positive, indicating that hypertension is a risk factor for DR (**Figure 3A**). The results of the forest plot implied that the SNP points for both MR Egger and IVW methods were on the right, supporting the notion that hypertension is a risk factor for DR (**Figure 3B**). The funnel plot depicted that MR is consistent with Mendel’s second law of random grouping (**Figure 3C**). The results of sensitivity analysis demonstrated that the *Q* value of this analysis was 0.057, which was greater than 0.05 without heterogeneity, and the *p*-value of horizontal pleiotropy was 0.621, indicating that there was no horizontal multi-effect. The forest plot of the leave-one-out method indicated that all error lines were to the right of 0, indicating that there were no points of deviation and that hypertension was a risk factor for DR (**Figure 3D**).

**Figure 4:**
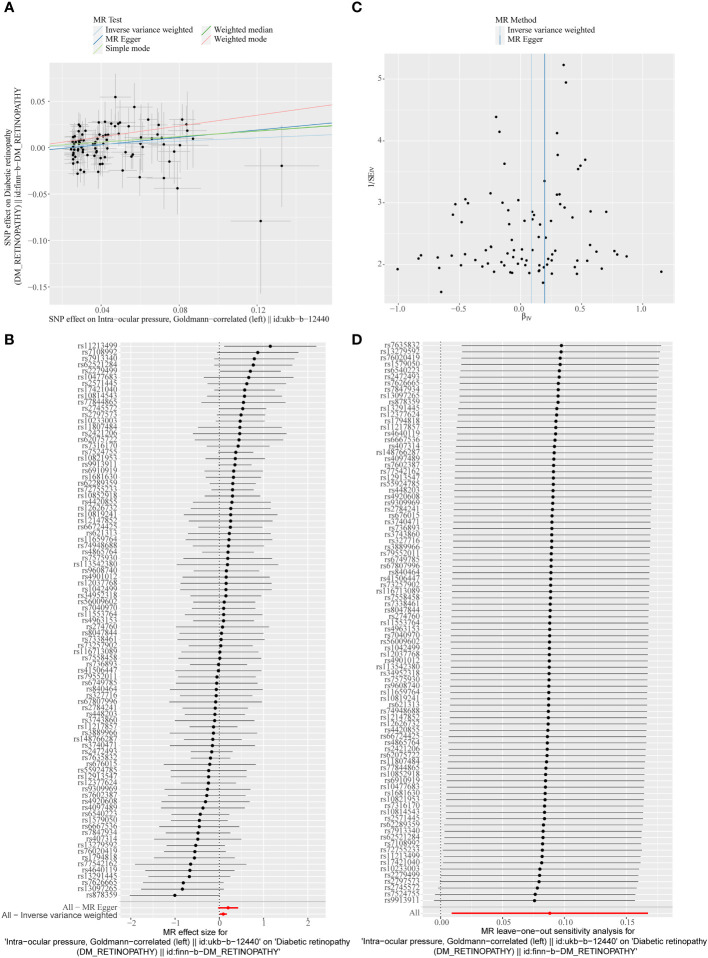
**(A)** Scatter plots of the MR analyses for the association of intraocular pressure (IOP) and the risk of DR. **(B)** Forest plot of MR results of IOP effect on DR. **(C)** Funnel plots of MR estimates for the effect of IOP on the risk of DR. **(D)** Leave-One-Out plot for sensitivity test for the effect of IOP on DR.

A total of 95 SNPs at the genome-wide significance level were selected as IVs for IOP-DR (**Supplementary Table 2**). Similarly, IOP was causally associated with DR, and IOP was a risk factor for DR (*p* = 0.029, OR = 1.090). The result of the weighted median method was similar to that of IVW (*p* = 0.013, OR = 1.160, **Figure 2**). The scatter plot suggested that the slope of the lines for all five methods was positive, suggesting that IOP is a risk factor for DR (**Figure 4A**). The results of the forest plot indicated that the SNP points for both MR Egger and IVW were on the right, supporting the notion that IOP is a risk factor for DR (**Figure 4B**). The funnel plot depicted that MR is consistent with Mendel’s second law of random grouping (**Figure 4C**). The results of sensitivity analysis implied that the *Q* value of this analysis was 0.052, which was greater than 0.05 without heterogeneity, and the *p*-value of Horizontal pleiotropy was 0.311, indicating that there was no horizontal multi-effect. The forest plot of the leave-one-out method demonstrated that all error lines were to the right of 0, indicating that there were no points of deviation and that IOP was a risk factor for DR (**Figure 4D**).

### 3.2 Reverse univariable MR analysis of DR, hypertension, and IOP

**Figure 5:**
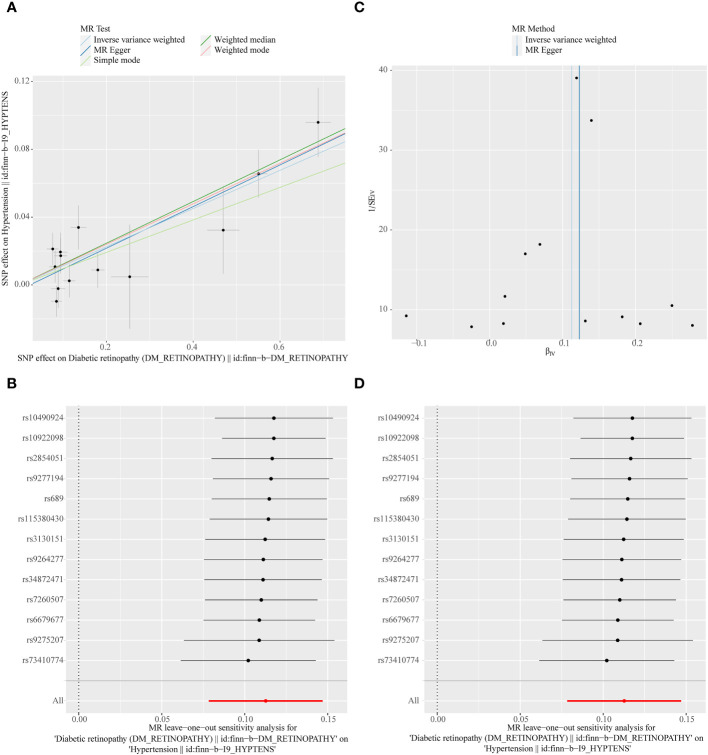
**(A)** Scatter plots of the MR analyses for the association of DR and the risk of hypertension. **(B)** Forest plot of MR results of DR effect on hypertension. **(C)** Funnel plots of MR estimates for the effect of DR on the risk of hypertension. **(D)** Leave-One-Out plot for sensitivity test for the effect of DR on hypertension.

In the primary analysis, a total of 14 SNPs at the genome-wide significance level were selected as IVs for DR-hypertension (**Supplementary Table 3**). The results of reverse univariable MR analyses suggested a causal relationship between DR and hypertension and DR as a risk factor for hypertension (*p* = 1.27×10^-10^, OR = 1.119, **Table 1**). The weighted median method result was similar to that of IVW (*p* = 2.72×10^-9^, OR = 1.131, **Table 1**). The scatter plot suggested that the slope of the lines for all five methods was positive, indicating that DR is a risk factor for hypertension (**Figure 5A**). The results of the forest plot implied that the SNP points for both MR Egger and IVW methods were on the right, supporting the notion that DR is a risk factor for hypertension (**Figure 5B**). The funnel plot depicted that MR is consistent with Mendel’s second law of random grouping (**Figure 5C**). The results of sensitivity analysis demonstrated that the *Q* value of this analysis was 0.250, which was greater than 0.05 without heterogeneity, and the *p*-value of horizontal pleiotropy was 0.598, indicating that there was no horizontal multi-effect. The forest plot of the leave-one-out method indicated that all error lines were to the right of 0, indicating that there were no points of deviation and that DR was a risk factor for hypertension (**Figure 5D**).

**Table 1:** Reverse Mendelian randomization (MR) results of diabetic retinopathy (DR) on hypertension.

Outcome	Exposure	Method	nSNP	b	SE	*p-*val	OR
finn-b-I9_HYPTENS	finn-b-DM_RETINOPATHY	MR Egger	13	0.122877	0.02608	0.000638	1.130745
finn-b-I9_HYPTENS	finn-b-DM_RETINOPATHY	Weighted median	13	0.122979	0.020678	2.72E-09	1.130861
finn-b-I9_HYPTENS	finn-b-DM_RETINOPATHY	Inverse variance weighted	13	0.112657	0.017517	1.27E-10	1.119248
finn-b-I9_HYPTENS	finn-b-DM_RETINOPATHY	Simple mode	13	0.095871	0.039243	0.030987	1.100617
finn-b-I9_HYPTENS	finn-b-DM_RETINOPATHY	Weighted mode	13	0.11957	0.020058	6.60E-05	1.127012

**Figure 6:**
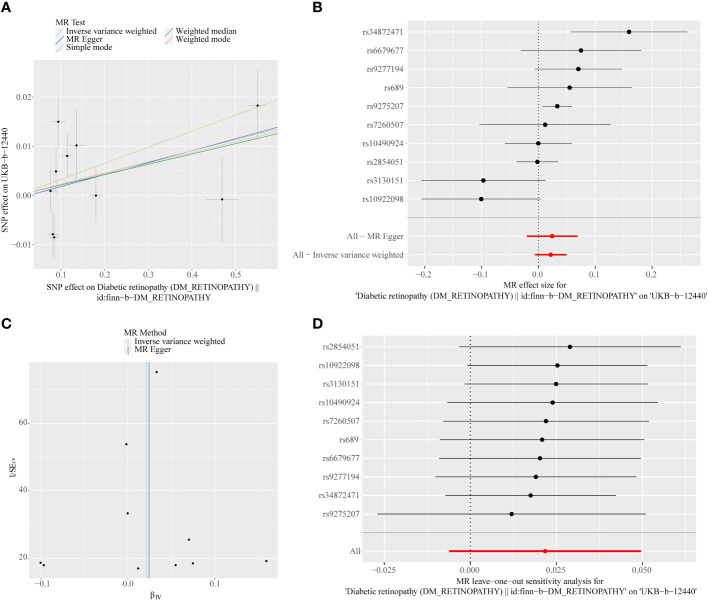
**(A)** Scatter plots of the MR analyses for the association of DR and the risk of IOP. **(B)** Forest plot of MR results of DR effect on IOP. **(C)** Funnel plots of MR estimates for the effect of DR on the risk of IOP. **(D)** Leave-One-Out plot for sensitivity test for the effect of DR on IOP.

A total of 11 SNPs at the genome-wide significance level were selected as IVs for DR-IOP (**Supplementary Table 4**). The *p*-values of the five MR methods were all greater than 0.05, indicating that there was no causal relationship between DR and IOP (**Table 2**). The combination of forest plots, scatter plots, and funnel plots supported the idea that there was no causal relationship between DR and IOP (**Figures 6A-C**). The results of sensitivity analysis showed that the reverse MR analysis was reliable (**Figure 6D**).

**Table 2:** Reverse MR results of DR on intraocular pressure (IOP).

Outcome	Exposure	Method	nSNP	b	SE	*p*-val	OR
finn-b-I9_HYPTENS	ukb-b-12440	MR Egger	10	0.049181	0.121216	0.685926	1.05041
finn-b-I9_HYPTENS	ukb-b-12440	Weighted median	10	−0.08831	0.045649	0.053045	0.915477
finn-b-I9_HYPTENS	ukb-b-12440	Inverse variance weighted	10	0.020739	0.042337	0.624231	1.020956
finn-b-I9_HYPTENS	ukb-b-12440	Simple mode	10	−0.16122	0.095152	0.093697	0.851105
finn-b-I9_HYPTENS	ukb-b-12440	Weighted mode	10	−0.1456	0.085836	0.093332	0.864504

### 3.3 Multivariable MR analyses

**Figure 7:**
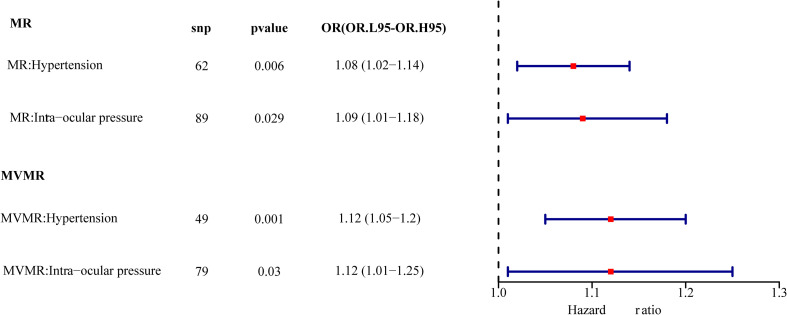
Univariate multivariate result forest map. The red square indicates that HR > 1, and the line segments on either side of the square are 95% CI for HR values.

Multivariable MR was used to determine how hypertension and IOP affected DR. After the screening of the IVs, 260 IVs (SNPs) associated with any exposure factor for hypertension or IOP were obtained. Of these 260 SNPs, 131 were unrelated to DR and were used as input for multivariate analysis. **Table 3** depicts that hypertension and IOP are still risk factors for DR, which have increased (*p* = 0.001, OR = 1.121, *p* = 0.030, OR = 1.124, **Figure 7**). Therefore, hypertension is still a risk factor for DR after the interference of IOP is eliminated; IOP is still a risk factor for DR after the interference of hypertension is eliminated.

**Table 3:** Multivariable MR analysis results of hypertension and IOP on DR.

Exposure	Outcome	nSNP	b	SE	*p*-val	OR
Hypertension || id:finn-b-I9_HYPTENS	Diabetic retinopathy (DM_RETINOPATHY) || id:finn-b-DM_RETINOPATHY	49	0.115	0.034	0.001	1.121
Intra-ocular pressure, Goldmann-correlated (left) || id:ukb-b-12440	Diabetic retinopathy (DM_RETINOPATHY) || id:finn-b-DM_RETINOPATHY	79	0.117	0.054	0.03	1.124

OR, odds ratio; nSNP, number of single-nucleotide polymorphisms; p-val, p values, p < 0.05 were considered statistically significant; b, the slope of the scatter plot; SE, standard error.

## 4 Discussion

This study is the first to explore the potential causal relationship between hypertension and IOP and the risk of DR via bidirectional MR analysis. It is confirmed at the genetic level that hypertension and IOP are independent risk factors of DR in forward MR, which will increase the risk of DR while changing simultaneously. Similarly, it is confirmed at the genetic level that DR is an independent risk factor of hypertension in reverse MR, which will increase the risk of hypertension while changing simultaneously. However, we did not find an association between DR and IOP from reverse MR analyses.

Numerous metabolic pathways are involved in the occurrence and development of DR; however, the interaction among various mechanisms remains elusive, necessitating additional research and clarification. It has been determined that various environmental, systemic, and ocular factors impact the incidence and risk of DR based on the study of clinical demographic characteristics and epidemiological and laboratory data. It has been confirmed that numerous risk factors, such as age, sex, hypertension, diabetes course, diabetic neuropathy, fasting blood glucose, serum total cholesterol, serum triglyceride, and HbA1c, are linked to the occurrence and development of DR; however, these risk factors only contribute to a small part of DR progression (21).

Epidemiological research on the relationship between DR and hypertension has been conducted for a long time. Although it is generally accepted that hypertension is associated with the risk of DR, there are conflicting views on this relationship in previous studies. Numerous studies have confirmed that hypertension can be a potential risk factor for DR. A cross-sectional study by Yang et al. revealed that the progression of diabetes, hypertension, and HbA1c are independent risk factors for DR formation in patients with diabetes but do not impact the diagnostic rate of DR in the general population (22). Some RCTs have proved that good regular blood pressure control is advantageous for the emergence of DR. Higher blood pressure level may be associated with the occurrence and progress of DR; however, mildly elevated hypertension does not significantly impact the development of DR (23). Meta-analysis based on clinical trials has demonstrated that low blood pressure can effectively reduce the risk of microvascular and macrovascular complications in DM (24, 25). Recently, an increasing number of researchers have revealed the close relationship between hypertension and DR via deep machine learning, the construction of risk prediction models, and the phenotypic and genetic analysis of large sample banks such as the UK Biobank (26–28). A large cohort study by Li et al. demonstrates that there is a non-linear dose–response association between higher SBP and an increased risk of microangiopathy in patients with diabetes (29). It remains elusive how the specific mechanism of hypertension affects the occurrence and development of DR. Some studies indicate that hypertension may affect microvascular and macrovascular diseases through hemodynamic changes and vascular endothelial growth factor-dependent pathways. Although certain studies continue to contest the multivariate regression analysis-based significant correlation between blood pressure and DR (30–33), the findings of this study are consistent with the majority of earlier publications (21, 34–37). The interaction between hypertension and DR is more evident, and for the first time, the clear causal inference that DR is the effect of hypertension is advanced. The mutual causal relationship between hypertension and DR was verified by bidirectional MR.

There are several publications on IOP and blood glucose; however, there is limited research that focuses on DR cohorts, and the previous studies’ findings regarding IOP and DR occurrence and development have not always been consistent. This study is the first to examine the relationship between IOP and DR by MR. Changes in IOP have reportedly been linked to DR in some earlier investigations (38–41). Jain et al. believed that low IOP was related to DR and proposed that low IOP or IOP changes would accelerate the formation of microhemangioma, which was the primary manifestation of DR (42). Other researchers have confirmed that IOP in individuals with DR progression exhibits a downward trend, which may be related to myopia, glaucoma, and arterial stenosis (41, 43, 44). Blanksma et al. observed that the IOP of children with DR was significantly higher than that of children with diabetes without retinopathy (45). This research’s conclusion is not widely accepted by scholars. Li et al. unexpectedly observed in a cross-sectional study in 2023 that IOP change is not an independent risk factor in DR progression, which is contrary to certain early research findings and the results of this study (31). Although a growing number of studies focus on the relationship between diabetes, DR, and IOP, up to now, studies directly correlating glaucoma with DR are still rare, and the results are inconsistent. A study conducted in India in 2020 revealed that glaucoma and DR are positively correlated in individuals with T2DM, and some other studies have confirmed this conclusion. A recent article released by Hong Kong scholars reveals that there is no significant correlation between DR and glaucoma, and stronger epidemiological and clinical evidence is needed to explain the relationship between DR and glaucoma (14, 46–48). Many scholars proposed the concept of mean ocular perfusion pressure (MOPP) based on the complex relationship between DR, blood pressure, and IOP. MOPP is calculated from mean arterial pressure (MAP) and IOP (MOPP = 2/3 MAP-IOP). Many studies have reported that MOPP is related to the occurrence and development of DR; however, the relationship between high MOPP and low MOPP and DR remains controversial. Some studies suggest that high MOPP is related to the development of DR, and low MOPP can prevent the development of DR. Other studies indicate that MOPP decreases with the deterioration of DR progression. However, Raman et al. demonstrated that there is no evidence to prove that there is a significant correlation between MOPP and DR through univariate and multivariate analysis. In a study published in 2023 by Chinese scholars, it was proposed that the increase in MOPP affected the occurrence of DR rather than affecting its progression (49–53). Currently, there is no widespread consensus on how IOP and DR are related. This study focuses on the genetic variation related to exposure factors. For the first time, other confounding factors were eliminated using the data from GWAS and MR analysis methods, and the causal relationship between IOP and DR is established. The change in IOP affects the risk of DR, which provides a clear direction for future research.

Additionally, this study is the first to evaluate the effects of hypertension and IOP on DR. Using multivariate Mendelian analysis, it was confirmed that hypertension and IOP were still independent risk factors after eliminating their mutual interference. In multivariate analysis, the risk factors of both increased; thus, we can speculate that when the two exposure variables of hypertension and IOP change simultaneously, the risk of DR would be greatly increased. Although previous studies have also analyzed the effect of combined blood pressure and IOP (MOPP, average intraocular perfusion pressure) on DR, the effect of simultaneous changes in blood pressure and IOP on DR risk has not been mentioned (54).

Compared with the traditional randomized controlled analysis, MR analysis has certain advantages. First, this study systematically examined the relationship between hypertension, IOP, and the risk of DR using univariate and multivariate MR analyses. Second, bidirectional MR was used to analyze the potential association between genetic variations related to hypertension, IOP, and DR, eliminating potential outliers and other confounding factors, and the reliability of the analysis results was verified by a series of sensitivity analyses. Third, before beginning the costly RCT, acquiring relatively reliable and accurate data through MR research will save time, energy, and research funds, thus enabling more valuable research.

However, there are still some shortcomings. The original analysis data in this study came from the Open GWAS database, which summarized and collected a large amount of GWAS data, but did not provide the original data or even sample information. Owing to objective data limitations, we unfortunately cannot provide baseline characteristics of participants. The MR study was conducted according to the GWAS database of the European population. Although the population differences were removed, it has not been confirmed whether it applies to other groups, such as those in Asia. The outcome of this MR study only suggests the causal relationship between hypertension, IOP, and DR; however, the potential mechanism is not specified, and the relationship between the increase and decrease in IOP and DR has not been addressed in this study. In addition, because of objective data limitations, we unfortunately cannot calculate the power of this study. Although the author is not an expert in MR analysis, he has extensive academic and research background in the field and has the appropriate knowledge and understanding.

In conclusion, this study confirmed at the genetic level that hypertension and IOP are independent risk factors of DR, which will increase the risk of DR while changing simultaneously. Numerous traditional observational studies, prospective studies, and considerably larger samples of GWAS data are still needed to test the causal relationship. Based on the previous research results and the conclusion of this MR analysis, clarifying the risk factors related to the occurrence of DR is crucial for early prevention, diagnosis, management, and extensive and effective screening of DR. We are supposed to delay or at least delay the onset and progression of DR through careful blood pressure and IOP control.

## Data availability statement

The original contributions presented in the study are included in the article/**Supplementary Material**. Further inquiries can be directed to the corresponding authors.

## Author contributions

X-FW: Methodology, Writing – original draft, Writing – review & editing. X-WZ: Resources, Supervision, Writing – review & editing. Y-JL: Resources, Writing – review & editing. X-YZ: Writing – review & editing. M-RS: Project administration, Resources, Supervision, Writing – review & editing. X-HS: Project administration, Resources, Supervision, Writing – original draft, Writing – review & editing. FJ: Project administration, Resources, Supervision, Writing – review & editing. Z-NL: Funding acquisition, Project administration, Supervision, Writing – review & editing, Resources.
